# Size-Dependent Ab Initio Atomistic Thermodynamics from Cluster to Bulk: Application to Hydration of Titania Nanoparticles

**DOI:** 10.1021/acs.jpclett.4c01531

**Published:** 2024-08-06

**Authors:** Miguel Recio-Poo, Ángel Morales-García, Francesc Illas, Stefan T. Bromley

**Affiliations:** † Departament de Ciència de Materials i Química Física & Institut de Química Teòrica i Computacional (IQTCUB), Universitat de Barcelona, c/Martí i Franquès 1-11, 08028 Barcelona, Spain; ‡ Institució Catalana de Recerca i Estudis Avançats (ICREA), Passeig Lluis Companys 23, 08010 Barcelona, Spain

## Abstract

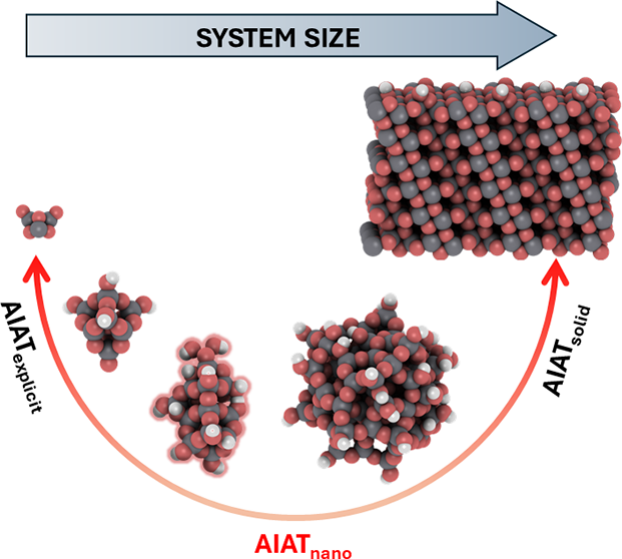

Ab initio atomistic thermodynamics (AIAT) has become an indispensable tool to estimate Gibbs free energy changes for solid surfaces interacting with gaseous species relative to pressure (*p*) and temperature (*T*). For such systems, AIAT assumes that solid vibrational contributions to Gibbs free energy differences cancel out. However, the validity of this assumption is unclear for nanoscale systems. Using hydrated titania nanoparticles (NPs) as an example, we estimate the vibrational contributions to the Gibbs free energy of hydration (Δ*G*_*hyd*_(*T*,*p*)) for arbitrary NP size and degree of hydration. Comparing Δ*G*_*hyd*_(*T*,*p*) phase diagrams for NPs when considering these contributions (AIAT_nano_) relative to a standard AIAT approach reveals significant qualitative and quantitative differences, which only become negligible for large systems. By constructing a size-dependent Δ*G*_*hyd*_(*T*,*p*) phase diagram, we illustrate how our approach can provide deeper insights into how nanosytems interact with their environments, with many potential applications (e.g., catalytic nanoparticles, biological colloids, nanoparticulate pollutants).

Computational modeling is increasingly playing a central role in the discovery and development of new materials that underly many technological advances. Here, approaches based on density functional theory (DFT) are widely employed due their capacity to calculate properties of materials accurately and rapidly. Formation of data sets of DFT-based calculations of thousands of compounds, and subsequent exploration using machine learning methods, can be employed to propose candidate materials with desirable characteristics for a class of applications.^1,2^ Still, however, the deliberate theoretical design of materials with structures and properties that are tailored for specific real life technological uses remains a significant challenge. To better address this problem, DFT-based models should account for the interaction of a material with its environment. This more realistic situation can be approximately captured for extended solid systems interacting with a gaseous phase by the *ab initio* atomistic thermodynamics (AIAT) approach.^3,4^ For such systems, AIAT makes simplifying assumptions (see below) that permit the application of standard DFT calculations. Decreasing system sizes to the nanoscale magnifies the complexity due to the emergence of a strong dependence of structures and properties on size and surface area. For nanosystems, the AIAT approximations used when modeling extended solids become more questionable. Accurately modeling the huge variety of important nanomaterials in realistic scenarios has thus been hindered by the theoretical challenges involved. Herein, to help address this issue, we propose a straightforward approach to estimate the thermodynamic stability of nanoscale structures in the presence of an interacting environment of gas-phase species.

Viable materials, irrespective of their size, should not only possess desirable characteristics but should also be synthesisable and sufficiently stable once made.^5^ These latter conditions are determined by both kinetics and thermodynamics. Kinetics can be studied to compare the rates of different synthetic routes followed to obtain a material from a given starting point. Thermodynamics is more fundamental as it pertains to whether such a synthesis is plausible under given conditions, regardless of the rate or route taken. The Gibbs free energy of formation, Δ*G*_*f*_(*T*,*p*), (i.e., the difference in Gibbs free energy between start and end points of a material’s synthesis at temperature *T* and pressure *p*) determines the favorability of the process. Phase diagrams derived from Δ*G*_*f*_(*T*,*p*) can thus serve as useful guides for predicting the stability of materials under realistic conditions.

From an experimental point of view, Δ*G*_*f*_(*T*,*p*) is typically viewed as being composed from the enthalpy of formation Δ*H*_*f*_(*T*,*p*) and an entropic term Δ*S*_*f*_(*T*,*p*), which are both functions of the heat capacity of the compounds involved in a formation reaction. The calorimetry experiments needed to accurately measure heat capacities of bulk materials are intricate and time-consuming,^6^ and become even more challenging for nanomaterials.^7^ The possibility to theoretically predict Δ*G*_*f*_(*T*,*p*) values for materials of arbitrary size with reasonable accuracy is thus very attractive. From a computational view, a standard DFT calculation of the internal energy of a chemical system at 0 K typically provides by far the most significant contribution to Δ*G*_*f*_(*T*,*p*). In contrast, explicitly calculating contributions to Δ*G*_*f*_(*T*,*p*) which depend on all relevant degrees of freedom (e.g., vibrations and atomic configurations) before and after a formation process, is relatively highly computationally demanding. For systems composed of hundreds or thousands of atoms explicit calculations of these contributions quickly become practically intractable. Although these contributions are typically much smaller than the 0 K internal energy, importantly, they can often be the deciding factor when assessing differences in Δ*G*_*f*_(*T*,*p*) for two competing processes.

For extended surfaces, DFT calculations have thus mainly been used to predict properties at 0 K in vacuum due to the high computational cost of explicitly evaluating all terms in Δ*G*_*f*_(*T*,*p*) for more realistic scenarios. However, under certain simplifying circumstances, Δ*G*_*f*_(*T*,*p*) values for extended solid surfaces interacting with a gaseous environment can be reasonably estimated by 0 K calculated energy differences of the solids plus thermodynamical properties of the gas phase molecules assumed to behave as an ideal gas.^8^ Here, Δ*G*_*f*_(*T*,*p*) values relate to the formation energies of systems where different proportions of gaseous molecules have adsorbed on the solid surface. With such an AIAT approach, one typically assumes that (i) the vibrational entropy contribution from surface atoms is the same for the clean and covered surface, and (ii) each system is dominated by very few low energy configurations. Then, for changes of coverage, the vibrational contributions to Δ*G*_*f*_(*T*,*p*) for the surfaces cancel out and the configurational entropic changes are negligible. The vibrational contribution of the adsorbates (typically the zero point energy – ZPE) can also be included in such calculations for a moderate extra computational cost. Here, the Δ*G*_*f*_(*T*,*p*) values become changes in enthalpies of the solid phase plus relevant changes due to gas phase (and sometimes adsorbed) species. Thus, Δ*G*_*f*_(*T*,*p*) values become accessible based on standard 0 K DFT calculations including normal modes of molecular species.

This general AIAT approach to extended solids (hereafter referred to as AIAT_solid_) has been widely used to predict the state of single-crystal metal surfaces in contact with a gaseous phase for a range of values of *T* and *p*.^3,4,9,10^ Generally, the AIAT approach relates to the use of 0 K calculated results to estimate Δ*G*_*f*_(*T*,*p*) values for solid systems in the presence of a reservoir of interacting chemical species.

For much smaller systems composed of up to a few tens of atoms interacting with a gaseous environment, one can use 0 K DFT calculations and standard statistical thermodynamics to directly calculate all relevant terms that contribute to Δ*G*_*f*_(*T*,*p*). An AIAT description of such a system does not rely on the simplifying assumptions used in the treatment of solid surfaces but is derived from explicit calculation of all relevant contributions to the partition function for all parts of the system (e.g., translational, rotational, vibrational, ZPE). This AIAT_explict_ methodology has been applied to calculate Δ*G*_*f*_(*T*,*p*) for numerous clusters reacting with gaseous environments (e.g., Mg_*n*_ clusters with oxygen,^11^ Au_*n*_ clusters with CO^12^ and (TiO_2_)_*n*_ and (SiO_2_)_*n*_ clusters with water vapor^13^).

Between small molecular scale clusters and extended macroscopic solids lie nanosized systems. Here, we find several important classes of objects which can strongly interact with their environments (e.g., catalytic nanoparticles, biological colloids, nanoparticulate pollutants) for which a Δ*G*_*f*_(*T*,*p*)-based characterization could be highly instructive. These systems are generally too large to be computationally tractable using an AIAT_explicit_ approach. It is also often unclear if such systems are sufficiently large such that one can use the approximations inherent in an AIAT_solid._ approach. Herein, we propose a simple method to estimate Δ*G*_*f*_(*T*,*p*) values for nanosystems interacting with gas phase species. We thus provide a general AIAT approach (hereafter referred to as AIAT_nano_) that is tailored for the bridging system size regime between the molecular scale and extended solids. In this way, we demonstrate how to exploit the powerful AIAT method for a significantly extended range of system sizes. Our work also highlights the size-dependent limitations of the approximations used in the standard AIAT_solid_ approach to solid surfaces. As our approach does not rely on any system specific properties, it should be straightforward to apply it generally to many nanosystems. By doing so, we hope that our AIAT_nano_ approach will yield new insights into how nanoparticulate systems interact with their environments with potentially wide-ranging implications.

As a specific example of our approach, we consider photoactive titania (TiO_2_) and its interaction with water molecules. Such a system is relevant to several technological applications (e.g., water purification, photoreduction of CO_2_, water splitting).^14−16^ Key to the AIAT_nano_ approach is the choice of reference systems. Ideally, for the lower size limit, these systems should be small enough to permit a AIAT_explicit_ treatment and large enough to exhibit typical nanoscale characteristics. Here, as an example, we mainly consider a NP system based on a 48 atom (TiO_2_)_16_ core structure cut from the bulk anatase crystal structure which we progressively hydrate while minimizing perturbations to its core structure. This system possesses the bulk-like stability and structure of larger anatase NPs (thus providing a natural limiting case for such systems) while allowing for an explicit calculation of all contributions to Δ*G*_*hyd*_(*T*,*p*) (i.e., the Gibbs free energy of hydration). We note that constraining the core structure of the NP to maintain a characteristic anatase-like structure leads to slightly different results in the final AIAT_nano_ approximation compared to the use of globally optimized (TiO_2_)_16_(H_2_O)_*m*_ structures^13^ (see S1 in the Supporting Information (SI)). As an example of a molecular scale system, we also consider a (TiO_2_)_4_(H_2_O)_*m*_ system for which globally optimized structures were used.^13^

For the large size limit, we use an extended anatase TiO_2_ (101) surface with different degrees of hydration. The surface model was represented by a periodically repeated slab consisting of six atomic layers of a (3 × 1) supercell, exposing the (101) surface on either side of the slab. Reciprocal space sampling at **Γ** point only was found to be sufficiently accurate.^17^ A 20 Å vacuum space between repeated slabs in the direction perpendicular to the surface was used avoid the artificial interslab interactions.

To compare our AIAT_nano_ approach with the standard AIAT_solid_ method, we consider a set of bipyramidal titania NP systems containing up to 500 atoms, namely (TiO_2_)_35_(H_2_O)_*m*_, (TiO_2_)_84_(H_2_O)_*m*_, and (TiO_2_)_165_(H_2_O)_*m*_. The titania cores of these NPs were obtained from top-down cuts of the bulk anatase crystal structure to expose facets of the most stable (101) surface^18−20^ To standardize our reported (TiO_2_)_*N*_ NP sizes, we use the diameter of a sphere containing *N* TiO_2_ units, where the volume of a single unit is taken from that in bulk anatase. This leads to diameters ranging from ∼2 nm (for the (TiO_2_)_35_ NP) to ∼4.3 nm (for the fully hydrated (TiO_2_)_165_ NP). These NPs are in the size regime for which direct DFT-based calculation of their vibrational frequencies would be extremely computationally expensive.

We assume that the hydration of all considered titania systems follows a mechanism where H_2_O dissociates upon adsorption and H and OH species interact with surface O and Ti atoms, respectively. This picture is supported by experiments on anatase NPs^21^ where progressive hydration proceeds from more reactive to less reactive regions (i.e., apical, equatorial, edge, and facet sites) until all coordinatively unsaturated atoms are covered. For extended (101) facets (e.g., on large crystalline anatase NPs) some molecular water adsorption will likely also occur along with dissociation.^22,23^ We note that molecular water adsorption is not likely to be prevalent on the relatively small NPs in our test set. Such a regime is also not relevant to the reported size-dependent example of our method where we focus only on the initial hydration step. The maximum degree of dissociative hydration in our (TiO_2_)_35_(H_2_O)_*m*_, (TiO_2_)_84_(H_2_O)_*m*_, (TiO_2_)_165_(H_2_O)_*m*_ NPs corresponds to *m* = 34, 62, and 98, respectively (i.e., (TiO_2_)_35_(H_2_O)_34_, (TiO_2_)_84_(H_2_O)_62_, and (TiO_2_)_165_(H_2_O)_98_). For the (TiO_2_)_16_(H_2_O)_*m*_ and (TiO_2_)_4_(H_2_O)_*m*_ systems, full dissociative hydration is reached with eight and four water molecules, respectively. Lastly, we considered a range of different degrees of hydration of our (101) anatase surface model. Figure 1 shows examples of titania systems considered, for: zero, minimal (i.e., one water molecule) and maximal degrees of hydration.

**Figure 1:**
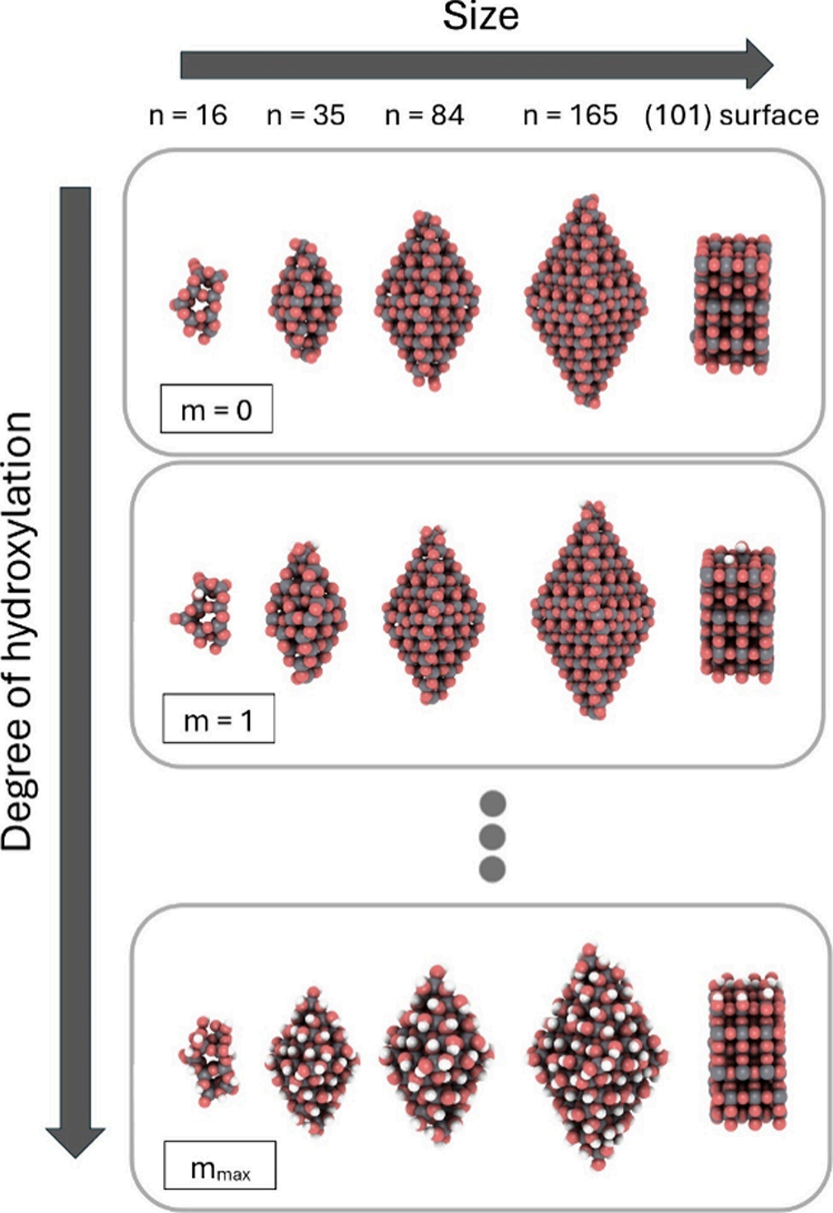
Examples of (TiO_2_)_*n*_(H_2_O)_*m*_ NP models and the anatase (101) surface supercell model used. White, gray, and red spheres denote H, Ti, and O atoms, respectively.

The structures of all as-cut anatase-based (TiO_2_)_*n*_(H_2_O)_*m*_ nanostructures and the (101) surface model were optimized using DFT-based calculations with the Perdew–Burke–Ernzerhof (PBE) exchange-correlation functional,^24^ as implemented in the FHI-aims code.^25^ A light-tier-1 numerical atom-centered orbital basis set was utilized in all cases, which provide results of triple-ζ plus polarization quality.^19^ The energy and force thresholds for the energy minimization and geometry optimization were fixed at 10^–5^ eV and 10^–4^ eV/Å, respectively.

The most computationally expensive part of explicitly evaluating Δ*G*_*hyd*_(*T*,*p*) involves the calculation of the vibrational degrees of freedom of the system. As this rapidly becomes intractable with increasing system size, we take (TiO_2_)_16_(H_2_O)_*m*_ as a reference system for which it is practical to explicitly calculate data to parametrize a temperature- and size-dependent *f*^*vib*^(*T*,*N*) function that considers contributions from all atoms of the system. As noted above, the size and properties of this reference system (e.g., anatase-like structure) are also chosen so that *f*^*vib*^(*T*,*N*) should provide a reasonable estimate of the vibrational contributions to Δ*G*_*hyd*_(*T*,*p*) for larger anatase-structured NPs. Although we expect that our (TiO_2_)_16_(H_2_O)_*m*_ NP system is a reasonable choice of an example to illustrate the expected type and magnitudes of expected contributions to Δ*G*_*hyd*_(*T*,*p*), how the specific form of *f*^*vib*^(*T*,*N*) depends on the chosen reference system will be studied in more detail in future work. We note that, ideally, the chosen reference system(s) for deriving *f*^*vib*^(*T*,*N*) should have structural/vibrational properties that are representative of NPs in the scalable regime (i.e., where NP properties scale in a regular manner with size).^26^

Figure 2 schematically compares our AIAT_nano_ approach with AIAT_explicit_ and AIAT_solid_ approaches used for smaller and larger systems, respectively.

**Figure 2:**
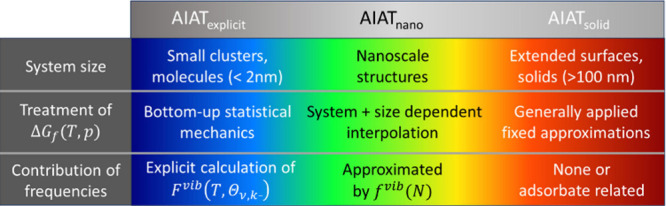
Scheme indicating three different system size regimes with respect to the respective AIAT-based computation of Δ*G*_*f*_(*T*,*p*). Blue and red, respectively, relate to the small and large regimes for which AIAT_explicit_ and AIAT_solid_ have been extensively applied. Our bridging interpolation approach for nanosized systems (AIAT_nano_) replaces the explicit computation of vibrational frequencies with the use of a temperature-dependent and *N*-dependent parametrized function, *f*^*vib*^(*T*,*N*), where *N* represents a generic system size variable.

To estimate Δ*G*_*hyd*_(*T*,*p*) for a given hydrated titania nanostructure at a temperature (*T*) and partial pressure of water vapor (*p*) at equilibrium we use:
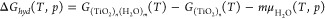
1where  is the chemical potential of the gas-phase water molecule as a function of *T* and *p*. In this way, Δ*G*_*hyd*_(*T*,*p*) estimates the free energy of hydration relative to the respective nonhydrated system. Once we have obtained the most stable degree of hydroxylation of a system under a range of conditions, the thermodynamic *p* versus *T* phase diagram can be derived. The vibrational contribution to Gibbs free energy, *F*^*vib*^, can be expressed as the sum of three terms:
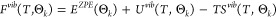
2where *U*^*vib*^ and *S*^*vib*^ are the vibrational contributions to the internal energy and entropy and *E*^*ZPE*^ corresponds to the zero-point energy (ZPE) contribution. Θ_*k*_ is the vibrational temperature, which depends on the normal mode frequencies (ν_*k*_). The standard analytical expressions for each term for a molecular system can be found in ref (27). *U*^*vib*^ and *S*^*vib*^ are dependent on *T* and are mainly influenced by lower frequencies, whereas *E*^*ZPE*^ is mainly governed by higher frequencies and is independent of *T*. In Figure S2 in the SI, we show how the explicitly calculated *F*^*vib*^(*T*,Θ_*k*_) at different temperatures varies with respect to frequency, ν_*k*_, for the (TiO_2_)_16_(H_2_O)_8_ system.

To circumvent the need for calculating all vibrational frequencies, we define an analytical expression, *f*^*vib*^(*N*,*T*), to approximate *F*^*vib*^. *f*^*vib*^(*N*,*T*) is derived to reproduce both contributions to *F*^*vib*^ for the hydrated (TiO_2_)_16_(H_2_O)_*m*_ NP system for different temperatures. We partition *f*^*vib*^(*N*,*T*) into two terms: *U*^*vib-ZPE*^ (combining temperature-dependent terms and ZPE) approximately accounting for energetic contributions and *S*^*vib*^, which estimates the entropic contributions:
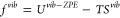
3We approximate these contributions using the following second order polynomial expressions:
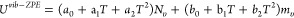
4
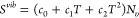
5where *N*_υ_ = 3(3*n* + *m*) – 6, which is total number of vibrations from all Ti and O atoms in a (TiO_2_)_*n*_(H_2_O)_*m*_ NP. Similarly, *m*_υ_ = 2*m* gives the total number of vibrations from the −O-H groups. The values of all coefficients are provided in the SI. The first term of *U*^*vib*-*ZPE*^ mainly captures the contribution from the low-frequency Ti–O vibrational modes connected to the temperature-dependent part, while the second term is mainly associated with the higher frequency vibrations (−OH) from the ZPE contribution. Although formally the ZPE contribution is temperature independent there is also a small contribution from the internal energy term from high −OH frequencies at high temperatures, which leads to a weak temperature dependence of the second term in eq 4. The *S*^*vib*^(*T*) term is only determined by lower frequency Ti–O vibrational modes. Figure S3 in the SI shows the evolution of both *U*^*vib-ZPE*^, and *T·S*^*vib*^ contributions to the Gibbs free energy for our (TiO_2_)_16_(H_2_O)_8_ NP with respect to *T*. As our simple derivation of *f*^*vib*^ is quite general with respect the types of vibrations expected in any (TiO_2_)_*n*_(H_2_O)_*m*_ NP, we assume that it provides a reasonable account of the vibrational contributions to the *G*_*hyd*_(*T*,*p*) for NPs of this type for any *n* and *m*. We also note that the thermodynamical terms involved in fitting *f*^*vib*^(*N*,*T*) are general to all nanoparticulate-adsorbate systems. We thus anticipate that fits like that used in eqs 4 and 5, with parameters adjusted to reflect the respective number/types of atoms and number/frequencies of vibrational modes, could be used for many nanosystems. We also note that although a simple polynomial fit was found to be adequate for this example system, other types of fit may be more appropriate for other nanosystems.

Using *f*^*vib*^ to estimate all the vibrational contributions to Δ*G*_*hyd*_(*T*,*p*), along with all other nonvibrational contributions calculated explicitly (e.g., 0 K internal energy, rotational translational entropic terms) we construct a AIAT_nano_ phase diagram showing the most stable (TiO_2_)_16_(H_2_O)_*m*_ compositions with for a range of temperatures and partial pressures of water vapor (see Figure 3b). Given the presumed applicability of Boltzmann statistics and the harmonic nature of molecular vibrations, we limit our temperature range to 100–1000 K, as in previous studies.^13,28^ We consider water partial pressures less than 1 × 10^5^ Pa allowing us to consider water as an ideal gas from around 450–500 K and above. For lower temperatures and higher pressures this is a more severe approximation, but its impact is confined to a small region of the phase diagrams only. Comparing the AIAT_nano_ phase diagram with one derived using AIAT_explict_ (see Figure 3a) we can see a reasonably good qualitative and quantitative match. In contrast, for the corresponding phase diagram derived by employing AIAT_solid_ (see Figure 3c), the agreement with the explicitly calculated phase diagram significantly worsens. Including explicitly calculated ZPE contributions (mainly from adsorbate vibrations) within an AIAT_solid_ approach (i.e., AIAT_solid_ + ZPE_ads_, see Figure 3d) only moderately improves the phase diagram with respect to the AIAT_explicit_ description. Conversely, including explicitly calculated non-ZPE temperature-dependent vibrational contributions to an AIAT_solid_ approach (see Figure 3e) significantly improves the phase diagram compared to the AIAT_explicit_ reference phase diagram. This comparison indicates that the temperature-dependent vibrational terms are the main reason for the differences between an AIAT_solid_ approach and a reference AIAT_explicit_ description.

**Figure 3:**
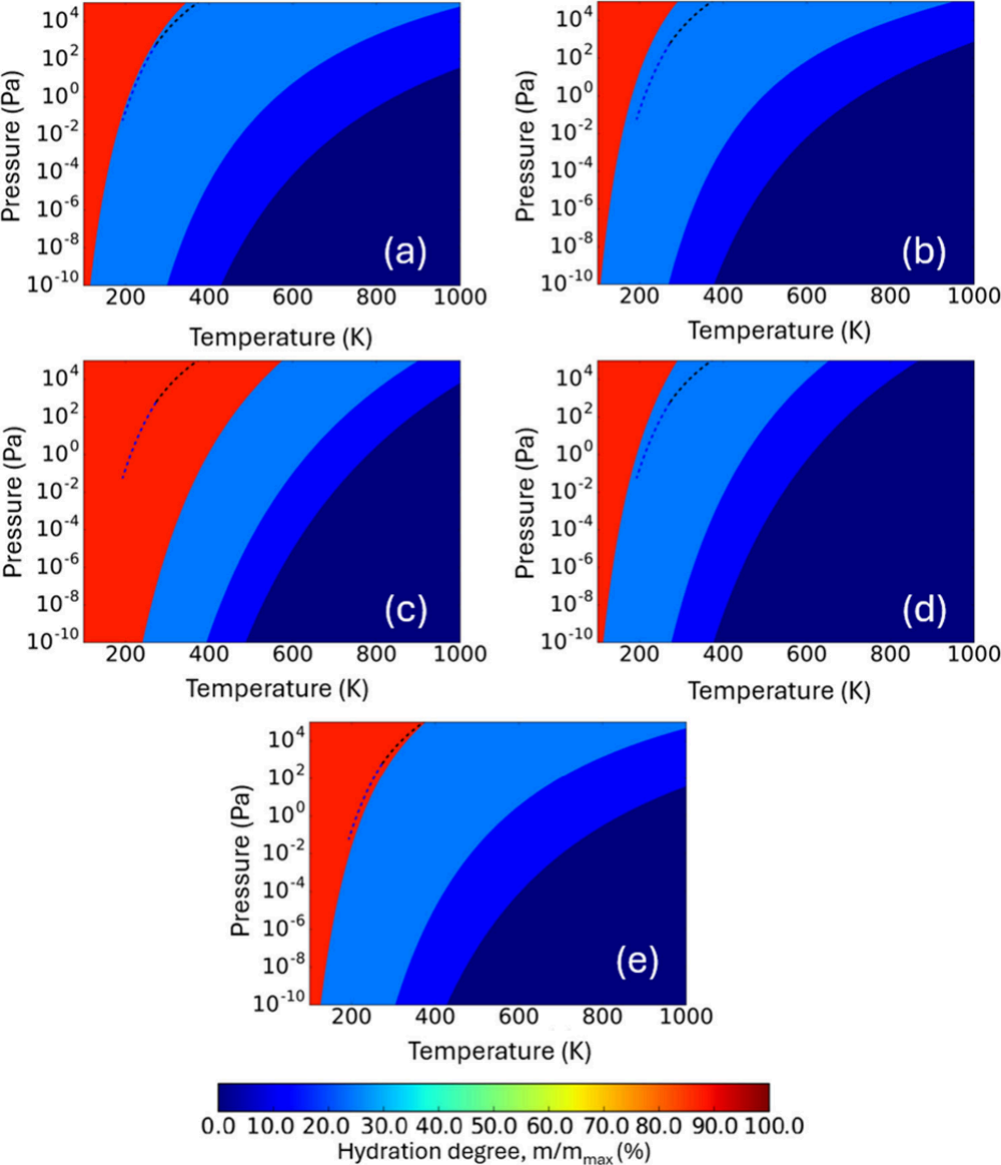
Thermodynamic *p*–*T* phase diagrams for the DFT-optimized (TiO_2_)_16_(H_2_O)_*m*_ NPs using different approaches to calculate differences in Δ*G*_*hyd*_(*T*,*p*): (a) AIAT_explicit_, (b) AIAT_nano_, (c) AIAT_solid_, (d) AIAT_solid_ + Δ*G*_ZPE_, and (e) AIAT_solid_ + Δ*G*_vib_. Blue and black dashed lines indicate the equilibrium vapor pressure with ice and liquid water, respectively. Within each phase diagram, each shaded region represents the most thermodynamically stable titania NP for a certain degree of hydration.

Using *f*^*vib*^, we can now estimate the vibrational contributions to Δ*G*_*hyd*_(*T*,*p*) for larger NPs in the scalable regime. We note that the size-dependency of Δ*G*_*hyd*_(*T*,*p*) values are largely dominated by 0 K energy differences which are considered in both AIAT_nano_ and AIAT_solid_ approaches. Generally, these energy differences are more pronounced for smaller systems than for larger systems, as hydration tends to perturb the former more than latter. From the definition of *f*^*vib*^ (see eqs 4 and 5), Δ*f*^*vib*^ values for a specific change in hydration degree do not explicitly depend on the size of the underlying titania system (*N*). This also implies that the differences in predictions of Δ*G*_*hyd*_(*T*,*p*) between AIAT_nano_ and AIAT_solid_ approaches, for any fixed conditions and for a specific hydration change, will not be size-dependent. However, such fixed shifts can still have a larger or smaller impact on thermodynamic crossovers for different NP sizes. To illustrate this, in Figure S4 in the SI, we show the temperature evolution of Δ*G*_*hyd*_(*T*,*p*) as predicted by AIAT_nano_ and AIAT_solid_ for system sizes of (TiO_2_)_4_(H_2_O)_*m*_ and (TiO_2_)_165_(H_2_O)_*m*_, for the hydration of the anhydrous systems by a single water molecule (for a water partial pressure of 1000 Pa). As expected, the temperature-dependent difference between the predicted variation in Δ*G*_*hyd*_(*T*,*p*) by AIAT_solid_ and AIAT_nano_ is the same for both system sizes. However, for each fixed system size, the predicted temperature at which Δ*G*_*hyd*_(*T*,*p*) changes sign (i.e., crosses the *x*-axis) is different for a AIAT_solid_ and AIAT_nano_ approaches. This change in sign signifies a change in the relative stability of the system with respect to hydration and is a key factor in defining the Δ*G*_*hyd*_(*T*,*p*) phase diagrams. Importantly, the size-dependency of the *T* and *p* conditions at which the predicted values of Δ*G*_*hyd*_(*T*,*p*) change sign will generally be different for a AIAT_nano_ approach with respect to a AIAT_solid_ approach. Consequently, the corresponding predicted Δ*G*_*hyd*_(*T*,*p*) phase diagrams for both approaches will be distinct and size-dependent.

In Figure 4, we show the predictions from an AIAT_nano_ approach as compared to AIAT_solid_ for Δ*G*_*hyd*_(*T*,*p*) phase diagrams for increasingly sized (TiO_2_)_35_(H_2_O)_*m*_, (TiO_2_)_84_(H_2_O)_*m*_ and (TiO_2_)_165_(H_2_O)_*m*_ model NPs. We note that for these systems explicit DFT-based calculation of all vibrational modes would be computationally very expensive, as compared to our (TiO_2_)_16_(H_2_O)_*m*_ reference system. For both considered AIAT approaches and all three NP sizes, all phase diagrams show an increasing preference for hydration as the temperature decreases for the full pressure range. Of the three considered NPs, as expected, the phase diagram for the smallest (TiO_2_)_35_(H_2_O)_*m*_ NP shows the largest difference with respect to the two AIAT approaches. For this size, differences in both the qualitative progression of the preferred degrees of hydration and the temperatures and pressures at which they are favored are seen. Slightly less dramatic differences between the two approaches are also observed for the larger two NPs. Here the qualitative description of the predicted Δ*G*_*hyd*_(*T*,*p*) phase diagrams is similar, but the quantitative agreement is often quite poor. This is particularly clear for higher pressures where, in line with Figure S4, we see that Δ*G*_*hyd*_(*T*,*p*) crossovers are predicted to occur at significantly higher temperatures in an AIAT_nano_ approach as compared to AIAT_solid_. Considering the similar comparison in Figure 3, these differences are likely mainly due to the inclusion of estimates of temperature-dependent vibrational contributions to Δ*G*_*hyd*_(*T*,*p*) in the AIAT_nano_ approach, rather than the effect of ZPE contributions.

**Figure 4:**
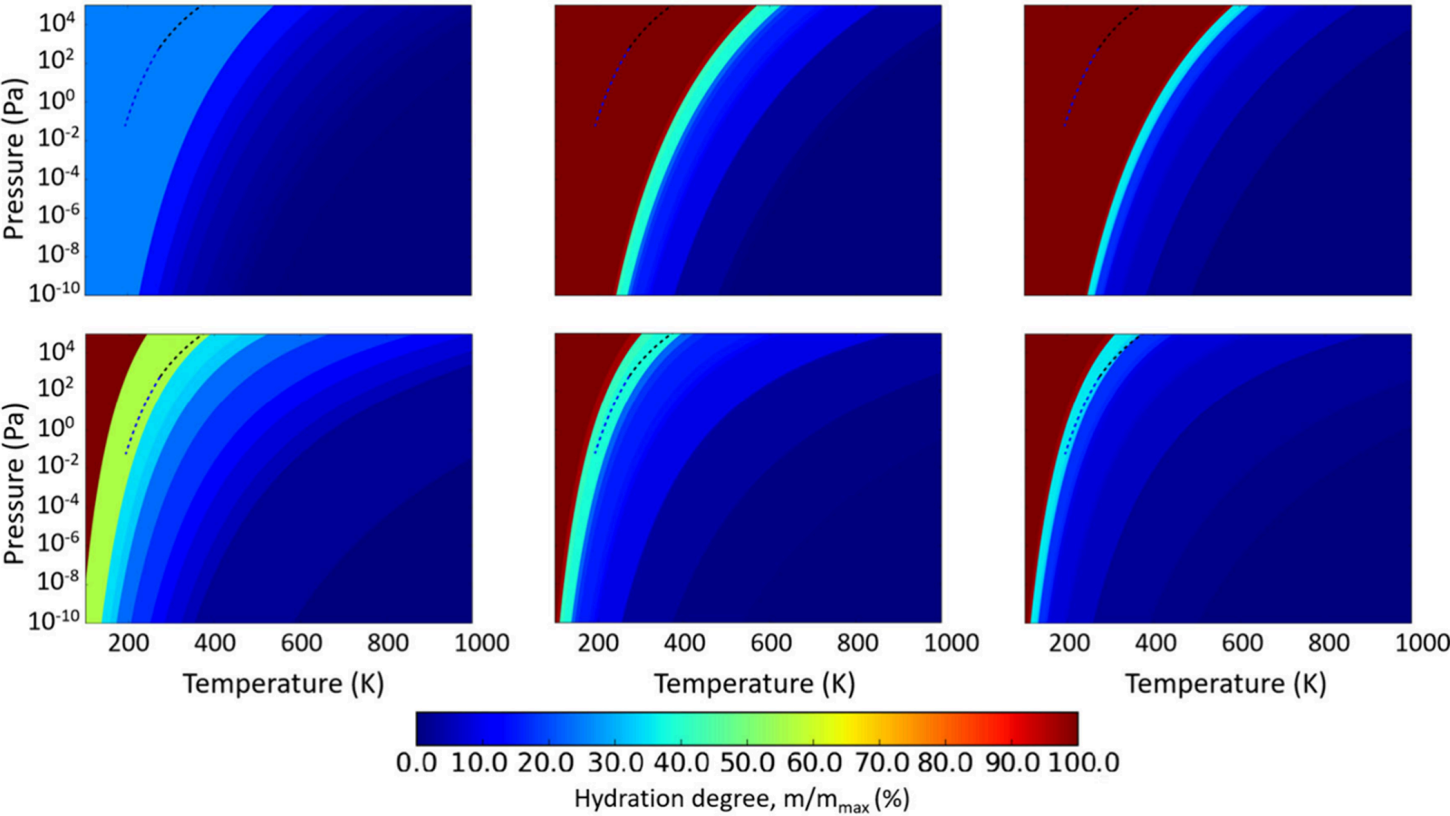
From left to right: predicted Δ*G*_*hyd*_(*T*,*p*) phase diagrams for the hydration of (TiO_2_)_35_(H_2_O)_*m*_ (left), (TiO_2_)_84_(H_2_O)_*m*_ (middle), and (TiO_2_)_165_(H_2_O)_*m*_ (right) obtained by using AIAT_solid_ (top) and AIAT_nano_ (bottom). Blue and black dashed lines indicate the equilibrium vapor pressure with ice and liquid water, respectively. Within each phase diagram, each shaded region represents the most thermodynamically stable titania NP for a certain degree of hydration.

As our AIAT_nano_ description is based on estimating total Δ*G*_*hyd*_(*T*,*p*) values from first-principles, it is independent of the approximations made in AIAT_solid._ and thus, does not necessarily converge to a AIAT_solid_ description with increasing size. However, we see in Figure 4 that the differences between the predicted Δ*G*_*hyd*_(*T*,*p*) phase diagrams from AIAT_nano_ and AIAT_solid_ appear to converge with increasing size. In Figure S5 we track the size-dependent AIAT_nano_ versus AIAT_solid_ differences in the predicted crossover temperature for the initial hydration onset. Here we can see that the highest sensitivity occurs for smaller system sizes (of a few hundred atoms) where the temperature difference can vary over more than 200 K for relatively small changes in system size. With increasing system size, we indeed see that this difference starts to converge to a constant value for which the large system size AIAT_nano_ predictions can range above and below the corresponding AIAT_solid_ predictions. We may expect that such differences should approach zero if the assumptions of AIAT_solid_ are taken to hold for the infinite sized limiting system. In such a case, we can use an AIAT_solid_ description of Δ*G*_*hyd*_(*T*,*p*) for an anatase bulk surface model as a size-limiting description for AIAT_nano_. Using this limit and an AIAT_nano_-based Δ*G*_*hyd*_(*T*,*p*) description for a set of finite systems, such as shown in Figure 4, we can interpolate between these two regimes (i.e., AIAT_nano_→ AIAT_solid_) to estimate the arbitrary size dependency any particular crossover.

Finally, to highlight a practical example of an AIAT_nano_→ AIAT_solid_ approach, in Figure 5 we show a size-temperature–pressure-dependent diagram predicting the thermodynamic conditions at which anatase NPs initially becomes hydroxylated (see section S6 of the SI). Again, we focus on the Δ*G*_*hyd*_(*T*,*p*) crossover contour dividing the anhydrous system and the system with a single water molecule adsorbed on it. Following a similar strategy to that described above (see also SI), we use the AIAT_nano_-predicted crossover contours from our (TiO_2_)_35_(H_2_O)_*m*_, (TiO_2_)_84_(H_2_O)_*m*_, and (TiO_2_)_165_(H_2_O)_*m*_ model NPs and the corresponding bulk AIAT_solid_-derived limiting contour derived for our anatase TiO_2_(101) surface model. In Figure 5 we highlight the contours corresponding to some selected NP diameters: (i) 1 nm, a size at which global optimization searches have established that noncrystalline anhydrous TiO_2_ NPs with quasi-spherical morphologies are the most energetically stable,^18^ (ii) 5 nm, which corresponds to the upper limit for TiO_2_ NPs to exhibit spherical-like NP morphologies,^29^ and (iii) 20 nm, which is close to the upper limiting NP size for relative thermodynamic stability of anatase with respect to rutile.^30^ For the 1 nm diameter crossover contour we are close in size to the bulk-mimicking (TiO_2_)_16_(H_2_O)_*m*_ NP that we employed to derive our *f*^*vib*^ function. Generally, below 5 nm in diameter, fully crystalline NPs tend to be less stable than amorphous quasi-spherical NPs, and thus we expect our predictions to tend to be more reliable for larger system sizes. We note that even for the crossover contour corresponding to crystalline anatase NPs with 20 nm diameters, the differences in *T* with respect to the bulk limiting case are still significant (20–90 K).

**Figure 5:**
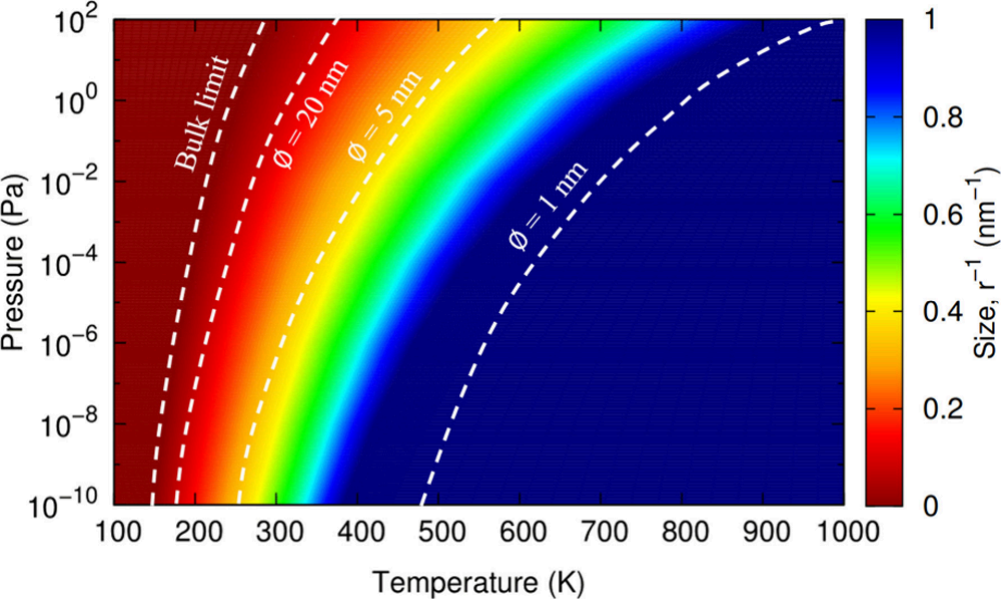
Size-dependency of the Δ*G*_*hyd*_(*T*,*p*) crossover contour for the initial hydration of an anhydrous anatase titania system by one water molecule. The colored regions correspond to different titania NP sizes. White dashed contours highlight selected NP sizes.

Overall, using the hydration of titania nanostructures as an example, we derive an analytical function, *f*^*vib*^, to approximate the Δ*G*_*hyd*_(*T*,*p*) values with respect to NP size and degree of hydration. *f*^*vib*^ accounts for the vibrational contributions to Δ*G*_*hyd*_(*T*,*p*) which avoids the explicit and computationally prohibitive calculation of system frequencies. As such, *f*^*vib*^ can be used to correct predicted differences in Δ*G*_*hyd*_(*T*,*p*) based on DFT-calculated 0 K total energies. The effect of *f*^*vib*^ on differences in Δ*G*_*hyd*_(*T*,*p*) diminishes with increasing system size but is still significant for nanostructures up to 10s of nm in diameter. In this way, the AIAT_nano_ approach can be used to calculate Δ*G*_*f*_(*T*,*p*) phase diagrams for nanosystems that are too large to use AIAT_explicit_ and too small to reliably employ AIAT_solid._ Our AIAT_nano_ approach thus bridges the gap between the small cluster size regime (diameters <2 nm) and extended surfaces and solids. As the AIAT_nano_ approach is not dependent on any system specific properties it is quite general and opens the door to a computationally efficient DFT-based treatment of nanoscale structures when interacting with their environment.

## Supporting Information Available

Comparison of a AIAT_explicit_ Δ*G*_*hyd*_(*T*,*p*) phase diagram for the globally optimized (TiO_2_)_16_(H_2_O)_*m*_ NP system compared with an AIAT_nano_ approach; vibrational contributions to Δ*G*_*hyd*_(*T*,*p*); fitting parameters for *f*^*vib*^(*N*,*T*); temperature dependence of vibrational terms in Δ*G*_*hyd*_(*T*,*p*); size-dependence of the initial hydration Δ*G*_*hyd*_(*T*,*p*) crossover contour; general size-dependency of AIAT_nano_ (PDF)
Transparent Peer Review report available (PDF)
