# Supplementary material for: Size-Dependent Ab Initio Atomistic Thermodynamics from Cluster to Bulk: Application to Hydration of Titania Nanoparticles
Source: J Phys Chem Lett. 2024 Aug 6;15(32):8240–7. doi: 10.1021/acs.jpclett.4c01531 (PMC11331519; doi:10.1021/acs.jpclett.4c01531)
Supplement: Supplementary file 1 — jz4c01531_si_001.pdf [file jz4c01531_si_001.pdf]

Size-dependent Ab Initio Atomistic Thermodynamics from Cluster to Bulk: Application to Hydration of Titania Nanoparticles

Miguel Recio-Poo,[a] Ángel Morales-García,*[a] Francesc Illas,[a] Stefan T. Bromley,*[ab]

[a]Departament de Ciència de Materials i Química Física & Institut de Química Teòrica i Computacional (IQTCUB), Universitat de Barcelona, c/Martí i Franquès 1-11, 08028 Barcelona, Spain

[b]Institució Catalana de Recerca i Estudis Avançats (ICREA), Passeig Lluis Companys 23, 08010 Barcelona, Spain

*Corresponding authors: s.bromley@ub.edu, angel.morales@ub.edu

S1. Comparison of an AIAT$_{explict}$ $\Delta G_{hyd}(T,p)$ phase diagram for the globally optimised $(TiO_2)_{16}(H_2O)_m$ NP system compared with a AIAT$_{nano}$ approach

S2. Vibrational contributions to $\Delta G_{hyd}(T,p)$

S3. Fitting parameters for $f^{vib}(N,T)$

S4. Temperature dependence of vibration-dependent terms in $\Delta G_{hyd}(T,p)$

S5. Size-dependence of the initial hydration $\Delta G_{hyd}(T,p)$ crossover contour

S6. General size-dependency of AIAT$_{nano}$ *vs* AIAT$_{solid}$

S1. Comparison of an AIAT$_{explicit}$ $\Delta G_{hyd}(T,p)$ phase diagram for the globally optimised $(TiO_2)_{16}(H_2O)_m$ NP system compared with a AIAT$_{nano}$ approach

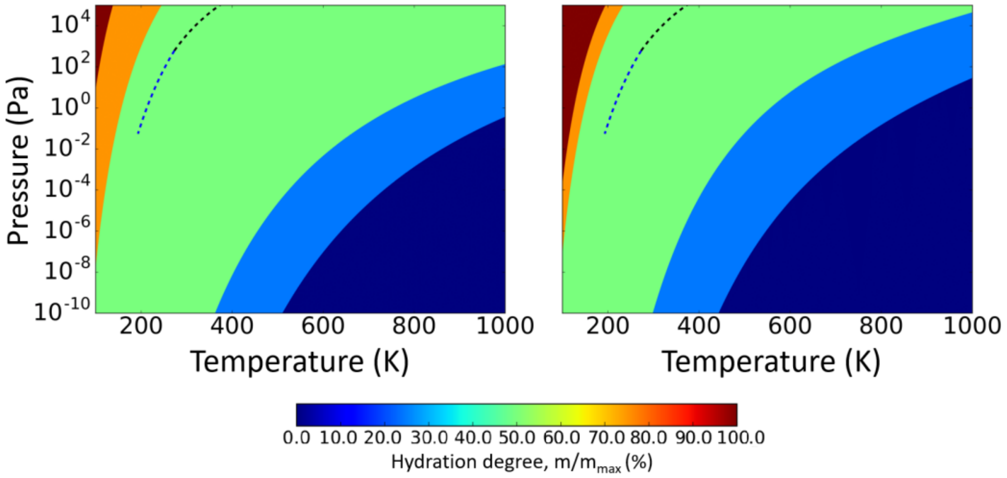

Figure S1. Thermodynamic $\Delta G_{hyd}(T,p)$ phase diagrams for the globally optimized $(TiO_2)_{16}(H_2O)_m$ NPs with all vibrational frequencies explicitly considered (i.e. via a AIAT$_{explicit}$ approach, left), and estimated using the f^{vib} function (i.e. via a AIAT$_{nano}$ approach, right). In the main text we employ $(TiO_2)_{16}(H_2O)_m$ NPs for which the core titania structure is constrained to keep close to the original cut from the anatase crystal.

S2. Vibrational contributions to $\Delta G_{hyd}(T,p)$

The vibrational contribution to Gibbs free energy, F^{vib}, can be expressed as the sum of three terms:

$$F^{vib}(T,\Theta_k) = E^{ZPE}(\Theta_k) + U^{vib}(T,\Theta_k) - TS^{vib}(T,\Theta_k) \quad (1)$$

where $U^{vib}$and S^{vib} are the vibrational contributions to the internal energy and entropy and E^{ZPE} corresponds to the zero-point energy (ZPE) contribution. Θ_k is the vibrational temperature, which depends on the frequencies (ν_k). Using our $(TiO_2)_{16}(H_2O)_8$ NP as an example, we explicitly compute the harmonic vibrational frequencies of the system and evaluate the $F^{vib}(T,\Theta_k)$. In Figure S2 we plot $F^{vib}(T,\Theta_k)$ with respect to vibration frequency and temperature (T).

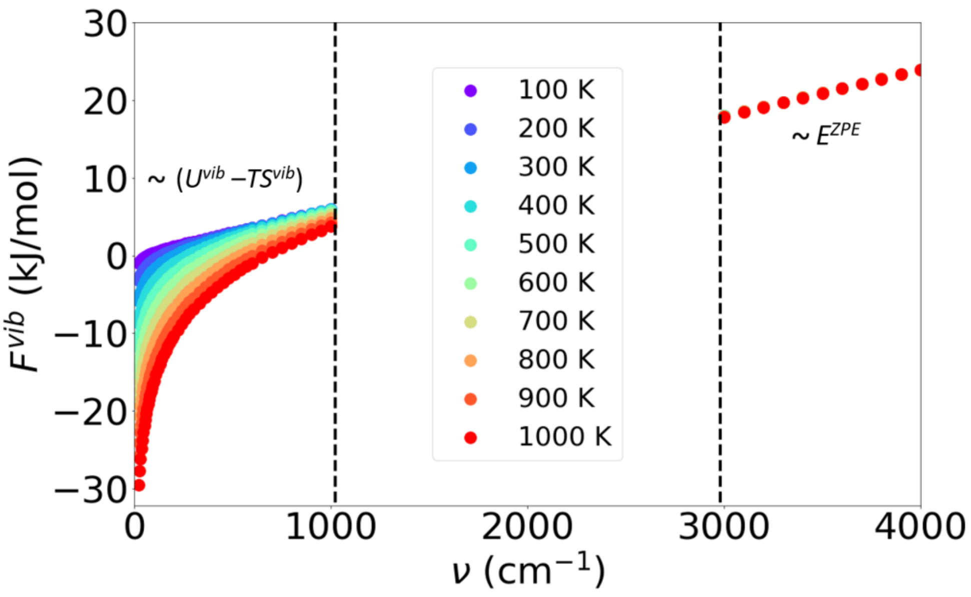

Figure S2. F^{vib} with respect to vibrational frequency (ν) at different temperatures calculated for the $(TiO_2)_{16}(H_2O)_8$ NP. The dominant influence of the temperature-dependent terms (lower frequencies) and the ZPE term (higher frequencies) are clearly differentiated.

Two distinct contributions to $F^{vib}(T, \Theta_k)$ are clearly seen. The first one at lower frequencies is almost totally defined by the temperature dependent terms and relates to the modes of the bonded Ti-O titania framework. The second contribution for higher frequencies, mainly corresponding to the temperature-independent ZPE term, is dominated by the vibrational modes of the pendant -OH groups.

S3. Fitting parameters for $f^{vib}(N, T)$

$f^{vib}(N, T)$ is derived to reproduce F^{vib} (i.e. the vibrational contributions to $\Delta G_{hyd}(T, p)$) for hydrated $(TiO_2)_{16}(H_2O)_m$ NPs at different temperatures. We partition $f^{vib}(N, T)$ into two terms: $U^{vib\text{-}ZPE}$ (combining temperature-dependent terms and ZPE) approximately accounting for energetic contributions, and S^{vib}, which estimates the entropic contributions:

$$f^{vib} = U^{vib-ZPE} - TS^{vib} \ (2)$$

We approximate these contributions using the following second order polynomial expressions:

$$U^{vib-ZPE} = (a_0 + a_1 T + a_2 T^2)\, N_v + (b_0 + b_1 T + b_2 T^2)\, m_v (3)$$

$$S^{vib} = (c_0 + c_1 T + c_2 T^2)\, N_v \ (4)$$

where $N_{\upsilon} = 3(3n + m) - 6$, which is total number of vibrations from all Ti and O atoms in a $(TiO_2)_n(H_2O)_m$ NP. Similarly, $m_{\upsilon} = 2m$ gives the total number of vibrations from -O-H groups. The coefficients used are:

$$a_0 = 1.98\,;\; a_1 = 4.28 \cdot 10^{-3}\,;\; a_2 = 2.47 \cdot 10^{-6}$$

$$b_0 = 21.76\,;\; b_1 = 3.75 \cdot 10^{-5}\,;\; b_2 = 5.55 \cdot 10^{-7}$$

$$c_0 = 6.32 \cdot 10^{-4};\; c_1 = 2.42 \cdot 10^{-5};\; c_2 = -9.74 \cdot 10^{-9}$$

Where a_0 and b_0 are in kJ/mol, a_1, $b_1$and c_0 are in kJ/(mol·T), a_2, b_2, and c_1 are in kJ/(mol·T^2), and $c_2$is in kJ/(mol·T^3).

S4. Temperature dependence of vibration-dependent terms in $\Delta G_{hyd}(T, p)$

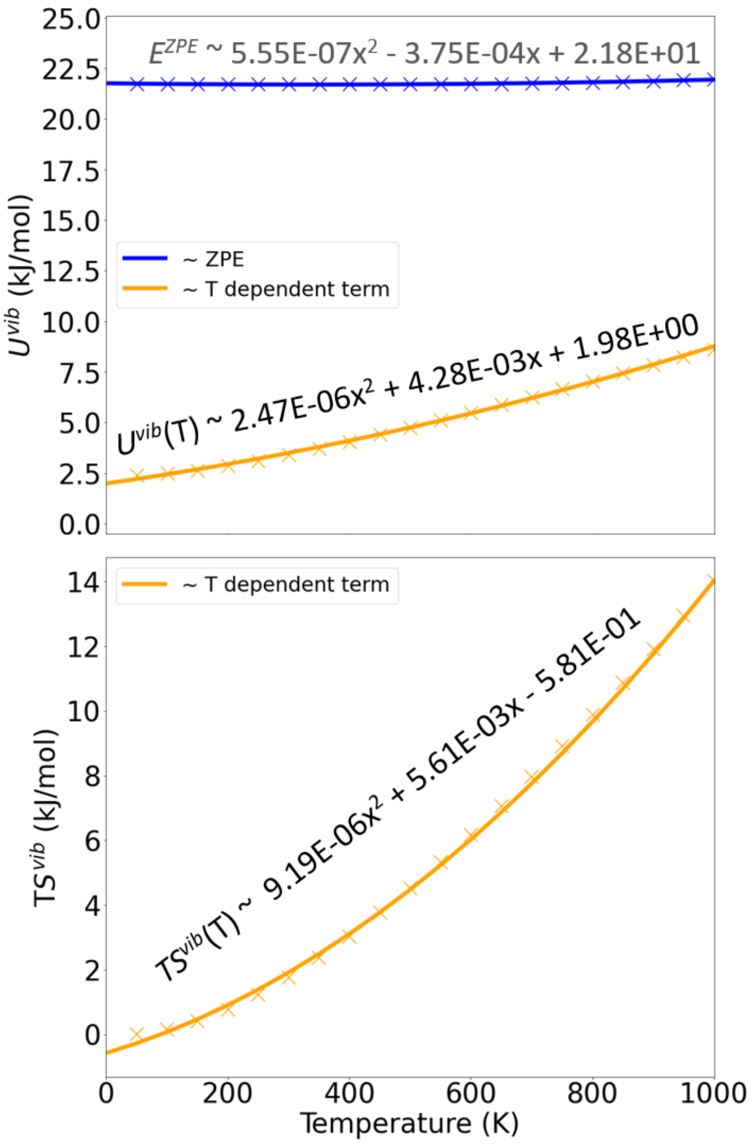

Figure S3. Explicitly calculated data points showing the temperature dependence of two terms making up the $U^{vib-ZPE}$ term (above) and the TS^{vib} term (below) for the $(TiO_2)_{16}(H_2O)_8$ NP. Solid lines follow the corresponding f^{vib} fitted expressions.

S5. Size-dependence of the initial hydration $\Delta G_{hyd}(T,p)$ crossover contour

In Figure S4 we show differences in $\Delta G_{hyd}(T,p)$ between that of anhydrous NPs and of the initially hydrated NPs at different temperatures (i.e. formally $\Delta\Delta G_{hyd}(T,p)$) for a water vapour partial pressure of 1000 Pa for two system sizes ($(TiO_2)_4$ and $(TiO_2)_{165}$) and for AIAT$_{nano}$ and AIAT$_{solid}$. For each system the constant shift between the AIAT$_{nano}$ and AIAT$_{solid}$ curves leads to a difference prediction in the crossover temperature (i.e. for $\Delta\Delta G_{hyd}(T,p) = 0$).

In figure S5 we extrapolate the tendencies in figure S4 to larger sizes to show the size-dependency of the predicted difference in crossover temperature as predicted by AIAT$_{nano}$ and AIAT$_{solid}$ approaches for five selected water partial pressures.

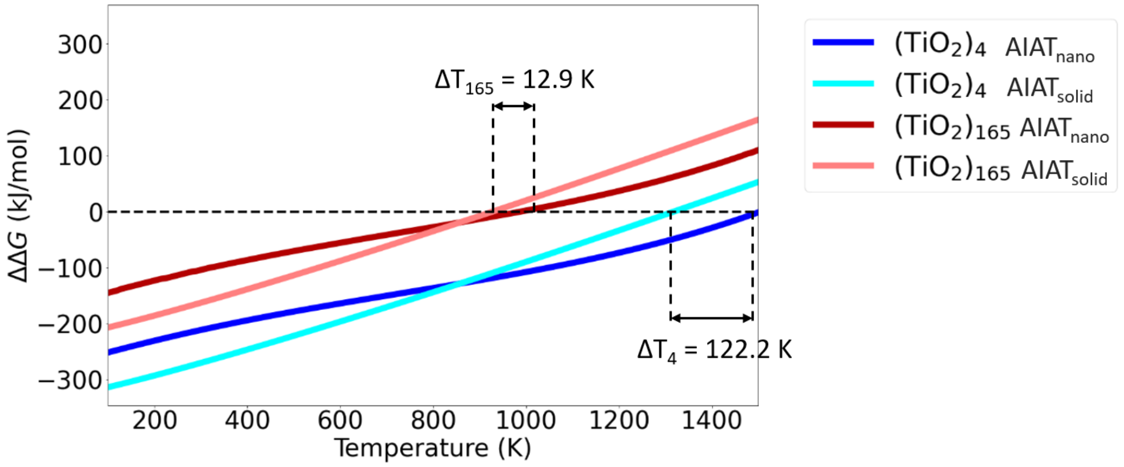

Figure S4. Temperature dependence of $\Delta\Delta G_{hyd}(T,p)$ following an AIAT$_{solid}$ approach (light coloured lines) and following our AIAT$_{nano}$ approach (dark coloured lines) with respect to the anhydrous ↔ initial hydration of a $(TiO_2)_4$ cluster (blue) and a $(TiO_2)_{165}$ NP (red) for a water vapour partial pressure of 1000 Pa. We highlight the AIAT$_{solid}$ versus AIAT$_{nano}$ temperature differences for this predicted crossover for the two systems considered.

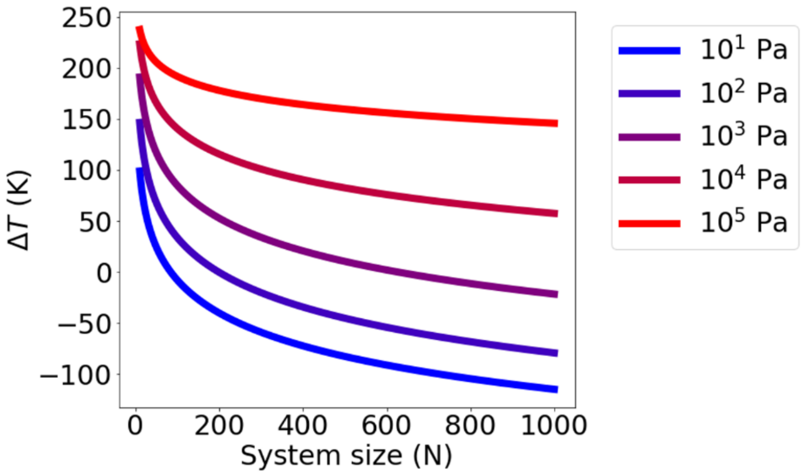

Figure S5. Differences in the predicted temperature of the initial $\Delta G_{hyd}(T,p)$ hydration crossover (i.e. between the anhydrous system and adsorption of a single water molecule) between AIAT$_{solid}$ and AIAT$_{nano}$ with respect to system size for selected water vapour partial pressure values.

S6. General size-dependency of AIAT$_{nano}$

Using f^{vib} derived $\Delta G_{hyd}(T,p)$ data for four model $(TiO_2)_n(H_2O)_m$ NPs (with *n* = 16, 35, 84 and 165) we found the anhydrous ↔ initially hydrated crossover temperature for a set of fixed pressures ranging from 2×10^{-10} to 200000 Pa. To do this we can follow the temperature for an incremental change in hydration by calculating differences in $\Delta G_{hyd}(T,p)$ between the system at the two degrees of hydration. For a fixed pressure, the condition $\Delta\Delta G_{hyd}(T,p) = 0$ then generally defines the temperature for a NP being at the crossover between the two degrees of hydration As an example, figure S6 shows the $\Delta\Delta G$ vs *T* evolution for a constant pressure of 1 Pa, for the anhydrous $(TiO_2)_{16}$ NP and the initially hydrated $(TiO_2)_{16}(H_2O)$ NP at different temperatures, yielding a limiting *T* value of ~250 K. Note that as $\Delta G_{hyd}(T,p)$ is calculated with respect to the anhydrous system (i.e. for which $\Delta G_{hyd}(T,p)$ = 0) then $\Delta\Delta G_{hyd}(T,p)$ is equivalent to $\Delta G_{hyd}(T,p)$ in this case.

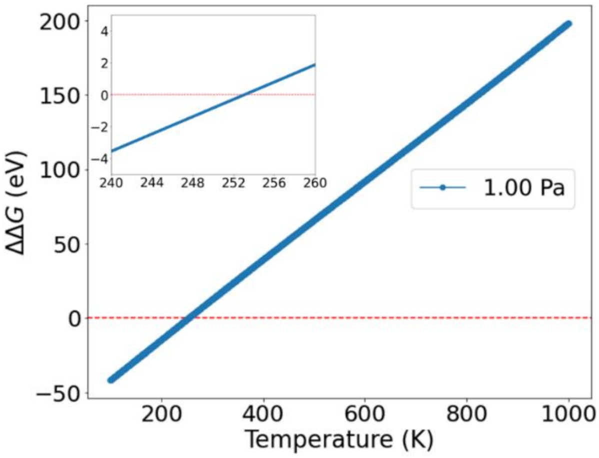

Figure S6. *ΔΔG vs T* plot for a constant 1 Pa pressure value for the $(TiO_2)_{16}(H_2O)_m$ NP, where ΔΔ*G* refers to the difference in $\Delta G_{hyd}(T,p)$ between the anhydrous NP and the initially hydrated NP.

For each constant pressure we thus obtain four points on a size-dependent isobar. We can also estimate the temperature of each isobar in the limit of infinite NP size by using extended periodic TiO_2(101) anatase surface model and a using a standard $AIAT_{solid}$ approach. By fitting these temperatures to an analytical function (see below), we can then estimate $\Delta G_{hyd}(T,p)$ crossover contours for any desired NP size.

To fit the size-dependent temperature profile for each isobar we used the fact that many size-dependency properties (*P*(*N*)) can be approximately fitted to a scaling law of the form[1,2]:

$$P(N) = P_{\text{bulk}} + a_1 N^{-\frac{1}{3}}, \qquad (4)$$

where P_{bulk} is a characteristic value of the property P for the chosen bulk phase. A generalisation of this relation leads to a *P(N)* size-dependence of the following form:

$$P(N) = a_0 + a_1 x + a_2 x^2 + a_3 x^3 + \ldots, (5)$$

where $a_0 = P_{bulk}$, $x = N^{-1/3}$ and the a_n values are (possibly *N* dependent) constants.

In our case the property *P*(*N*) is the size-dependent crossover temperature for each isobar (i.e. *T*(*n*), where *n* is the number of TiO_2 units and where the a_0 term corresponds to the bulk limiting *T* value). We find that good fits can be made for each isobar with using polynomials of degree two or three (see examples in Figure S7).

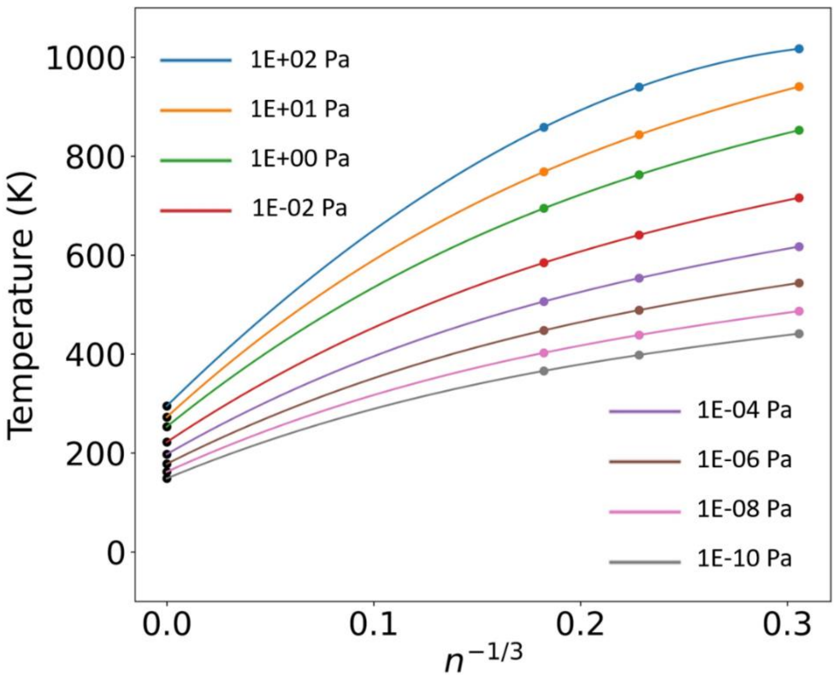

Figure S7. Temperature versus $n^{-1/3}$ plot showing the fitted isobars for a range of water vapour pressures. Where the temperature relates to the anhydrous-initially hydrated crossover temperature in each isobar and n corresponds to the number of TiO_2 units in the system.

References

(1) R. L. Johnston, R. L. Atomic and Molecular Clusters (Master's Series in Physics and Astronomy) ISBN 13: 9780748409310, Taylor & Francis Ltd **2002**.

(2) Lamiel-Garcia, O.; Cuko, A.; Calatayud, M.; Illas, F.; Bromley, S. T. Predicting size-dependent emergence of crystallinity in nanomaterials: titania nanoclusters versus nanocrystals. *Nanoscale* **2017**, *9*, 1049-1058.
