# Supplementary material for: Size-Dependent Ab Initio Atomistic Thermodynamics from Cluster to Bulk: Application to Hydration of Titania Nanoparticles
Source: J Phys Chem Lett. 2024 Aug 6;15(32):8240–7. doi: 10.1021/acs.jpclett.4c01531 (PMC11331519; doi:10.1021/acs.jpclett.4c01531)
Supplement: Supplementary file 2 — jz4c01531_si_002.pdf [file jz4c01531_si_002.pdf]

jz-2024-01531n.R1

Name: Peer Review Information for "Size-dependent Ab Initio Atomistic Thermodynamics from Cluster to Bulk: Application to Hydration of Titania Nanoparticles"

First Round of Reviewer Comments

Reviewer: 1

Comments to the Author

The paper "Size-dependent Ab Initio Atomistic Thermodynamics from Cluster to Bulk: Application to Hydration of Titania Nanostructures" reports an original discussion of how to determine the vibrational contributions to the free energy of adsorbate-covered surfaces for nanosystems of size intermediate between that of a small molecule and an extended surface. For these systems a direct calculation of all vibrational contributions to the free energy becomes practically intractable. The authors propose an approach to estimate the vibrational contributions to the free energy of nanostructures using water adsorption on TiO_2 nanoparticles as an example. This new approach is the major advance of the paper. The work is solid, the approach is novel, and the work deserves to be published in present form.

The only concern is related to the length of the paper and the depth of the discussion: 15 pages, 7 figures, are more appropriate for a regular article than for a letter. But this is something that has to be decided at the editorial level.

Reviewer: 2

Comments to the Author

First of all, the development of AIAT_nano method is very important to the field. As also pointed by the authors, the explicit AIAT is not computationally feasible for the nanoparticles including 100 – 500 or even more atoms while the cancellation of the vibrational free energy used in AIAT for the solid surface is not suitable for the nanoparticles where the vibrational contribution is considerable.

However, the AIAT_nano method proposed in this work is limited to one specific system, here the hydration of Ti_xO_y nanosystems. The method is also too straightforward by just fitting the parameters of the polynomial only using the data of nanoparticles when x = 16.

ALso, a fair comparison should be between the AIAT explicitly calculating the frequencies and the AIAT_nano they proposed.

More importantly, in the nanosystems, not only the vibrational contribution is significant but also the entropic contributions. It is really hard to say how the method proposed here matters in the real

application.

The generalization of the proposed method limits the use of the method to other nanosystems. The manuscript should also be improved by writing concise and precise.

I would like to recommend the current version might be suitable to Journal of Physical Chemistry C instead of the Journal of Physical Chemistry Letter.

Author's Response to Peer Review Comments:

Dept. de Ciència de Materials i Química Física &
Institut de Química Teòrica i Computacional
Universitat de Barcelona
C/ Martí i Franquès, 1
08028 BARCELONA (SPAIN)
Tel: +34 934039732
E-mail: **s.bromley@ub.edu**

Barcelona, July 17th, 2024

Dear Editor,

We thank the reviewers for their time and effort, and the points raised. Detailed responses to all comments are provided below and corresponding changes to the revised manuscript and/or Supporting Information are noted as necessary. All revisions to the text of the manuscript are marked in red.

All editorial requests concerning non-scientific changes to the manuscript and SI have also been implemented.

Sincerely,

Stefan T. Bromley (on behalf of all authors)

Reviewer 1

This paper represents a significant new contribution and should be published as is.

The paper "Size-dependent Ab Initio Atomistic Thermodynamics from Cluster to Bulk: Application to Hydration of Titania Nanostructures" reports an original discussion of how to determine the vibrational contributions to the free energy of adsorbate-covered surfaces for nanosystems of size intermediate between that of a small molecule and an extended surface. For these systems a direct calculation of all vibrational contributions to the free energy becomes practically intractable. The authors propose an approach to estimate the vibrational contributions to the free energy of nanostructures using water adsorption on TiO2 nanoparticles as an example. This new approach is the major advance of the paper. The work is solid, the approach is novel, and the work deserves to be published in present form.

We thank the reviewer for the positive evaluation of our work.

The only concern is related to the length of the paper and the depth of the discussion: 15 pages, 7 figures, are more appropriate for a regular article than for a letter. But this is something that has to be decided at the editorial level.

Indeed, this is in line with an editorial request. We have now moved two figures to the SI and reduced the main text to be of a more appropriate length for a letter (i.e. <4000 words excluding abstract, captions and references).

Reviewer 2

First of all, the development of AIAT_nano method is very important to the field. As also pointed by the authors, the explicit AIAT is not computationally feasible for the nanoparticles including 100 – 500 or even more atoms while the cancellation of the vibrational free energy used in AIAT for the solid surface is not suitable for the nanoparticles where the vibrational contribution is considerable.

We thank the reviewer for noting the importance of the objectives of our $AIAT_{nano}$ method.

However, the AIAT_nano method proposed in this work is limited to one specific system, here the hydration of TixOy nanosystems.

As mentioned in the abstract and main text, the hydration of titania nanoparticles is used only as an example of how to apply the $AIAT_{nano}$ method. We see nothing overly specific about this example that limits its more general application to many other nanosystems. We now make this point clearer in the revised text.

The method is also too straightforward by just fitting the parameters of the polynomial only using the data of nanoparticles when x = 16.

The method uses an analytical expression for the size/coverage-dependent vibrational contributions to the free energy (including the ZPE). For our example case we found this could be achieved by a polynomial fit. This is not a constraint of the approach, and other fits/expressions could be used for other systems. We used the $(TiO_2)_{16}(H_2O)_m$ system for this fitting procedure as it provides a model for which all relevant free energy terms could be calculated explicitly (see next point) but is large enough to exhibit similar tendencies to those found for larger nanosystems. Our study provides an example of how to apply the $AIAT_{nano}$ method. For the reported system and other systems, larger NPs or sets of NPs could be used for making an even more robust/general model. These points are now further clarified in the revised text.

Also, a fair comparison should be between the AIAT explicitly calculating the frequencies and the AIAT_nano they proposed.

We agree. This important comparison is shown for the $(TiO_2)_{16}(H_2O)_m$ system in figure 3 and discussed in the main text. This comparison also highlights the importance of using a reference system for which it is tractable to calculate all frequencies explicitly.

More importantly, in the nanosystems, not only the vibrational contribution is significant but also the entropic contributions. It is really hard to say how the method proposed here matters in the real application.

Our approach considers all vibrational contributions to the free energy – including entropic contributions. These entropic contributions are shown to be particularly important (see figure 3) and are shown well approximated by our approach. As such, we believe our approach would be particularly important in many real applications.

The generalization of the proposed method limits the use of the method to other nanosystems.

We see no reason why our example could not serve as a model for applying our approach more generally to many other nanosystems (also see above responses). We now highlight this point in the revised text.

The manuscript should also be improved by writing concise and precise.

We have reduced the main text to be more appropriate for a letter. We have also tried to further clarify the purpose of the current study (i.e. as an illustrative example of applying the method) and to better

describe our implementation so that it can be more easily used to guide applications to other nanosystems.

I would like to recommend the current version might be suitable to Journal of Physical Chemistry C instead of the Journal of Physical Chemistry Letter.

We hope that the above clarifications and our edits to the text of the manuscript convince the reviewer that our study is more suitable for publication in JPCLett.
